# Intervention effectiveness reducing disability stigma in sub-Saharan Africa: Systematic review

**DOI:** 10.4102/ajod.v15i0.1780

**Published:** 2026-01-12

**Authors:** Bhavisha Virendrakumar, Cathy Stephen, Emma Jolley, Vladimir Pente, Elena Schmidt

**Affiliations:** 1 Department of Policy and Programme Strategy, Sightsavers, Haywards Heath, United Kingdom; 2 Department of Policy and Programme Strategy, Sightsavers, Yaounde, Cameroon

**Keywords:** disability, stigma, intervention, effectiveness, systematic review

## Abstract

**Background:**

To reduce stigma, there is a need to understand where stigma exists, how it affects different populations, and what interventions have proven effective in reducing stigma.

**Objectives:**

To synthesise evidence on intervention effectiveness in reducing disability-related stigma in sub-Saharan Africa.

**Method:**

We conducted a comprehensive search of nine databases, supplemented by grey literature, references and expert consultations. Two authors screened, extracted and appraised studies. Interventions were categorised according to the Behaviour Change Wheel framework, and synthesised narratively from those with a low and medium risk of bias.

**Results:**

Out of 15 studies, eight studies (four each with low and medium risk of bias) reported positive effects, seven found education and training effective, either alone or with other interventions. One study observed positive effects from combining education with communication, enablement and persuasion. Of the five studies with mixed effects (medium risk of bias), four employed education and training alongside other interventions, and one combined education with modelling, persuasion, enablement and communication. Two studies (low risk of bias) reported null effects when combining education, training and service provision with other interventions.

**Conclusion:**

High-quality research on the impact of stigma interventions in sub-Saharan Africa is limited. Challenges include defining stigma, proving intervention effectiveness, and the varied target groups, settings, intervention types and metrics used to measure stigma change.

**Contribution:**

This study highlights the need for and provides the rationale for increased methodological rigour and theoretical grounding in the evaluation of stigma-reduction interventions, and full and transparent reporting of all results.

## Introduction

The World Health Organization (WHO) estimated that in 2021, 16% of the global population (1.3 billion people) lived with some form of disability, with women, older people, and people living in low- and middle-income countries (LMICs) being disproportionately affected. The United Nations Convention on the Rights of Persons with Disabilities (UNCRPD) describes persons with disabilities as:

[*T*]hose who have long-term physical, mental, intellectual or sensory impairments, which in interaction with various barriers may hinder their full and effective participation in society on an equal basis with others. (United Nations 2022:n.p.)

It includes people with hearing loss or deafness, learning difficulties, dementia and others. Although human immunodeficiency virus (HIV) or acquired immunodeficiency syndrome (AIDS) is not explicitly mentioned in the UNCRPD, it is increasingly recognised as a disabling condition because of its long-term health impacts and the exclusion experienced through stigma and discrimination (Elliott, Utyasheva & Zack 2009; Equality and Human Rights Commission 2010; UNAIDS 2017). International frameworks support this interpretation: guidelines from the Joint United Nations Programme on HIV or AIDS (UNAIDS), and the Office of the High Commissioner for Human Rights (OHCHR) affirm that anti-discrimination laws should protect individuals based on actual or perceived HIV status (Office of the United Nations High Commissioner for Human Rights; UNAIDS 2024). Accordingly, this review includes HIV or AIDS as one of the disabling conditions.

People with disabilities experience multiple disadvantages across health, education and social domains (Kuper & Heydt 2019; World Health Organization 2011). Stigma and negative social attitudes are recognised as major barriers to inclusion (Rohwerder 2018).

Link and Phelan (2001) define stigma as the co-occurrence of labelling, stereotyping, separation, status loss and discrimination, emphasising that power is central to its operation. Stigma-related harmful consequences include social exclusion, reduced access to services and increased social inequalities (Link & Phelan 2001).

Several frameworks have been developed to explore health- and disability-related stigma (Goffman 2009; Scambler 1986; Stangl et al. 2019; Weiss 2008). Goffman’s theory, introduced in the 1960s, distinguished between two forms of stigma: felt stigma, referring to the internal experience of feeling discriminated against; and enacted stigma, which involves acts of discrimination and stigmatisation (Goffman 2009). Scambler (1986) introduced the ‘Hidden Distress Model’ in his research on epilepsy, arguing that felt stigma often has a greater disruptive impact on the lives of people with epilepsy than enacted stigma (Scambler 1986). Weiss (2008) expanded on this model through studies of stigma related to neglected tropical diseases. These studies introduced the concepts of anticipated and internalised stigma and extended the approach to develop tailored interventions aimed at reducing stigma. The latter describes a process in which individuals with a stigmatised condition internalise negative stereotypes and project them onto themselves, reinforcing feelings of social exclusion (Weiss 2008). The approach is useful for understanding how different types of stigma relate to one another, and for identifying interventions that may contribute to their reduction. Stangl et al. (2019) developed a framework for understanding stigma across various health conditions, initially for HIV or AIDS, but later applied to leprosy, epilepsy, mental health, cancer and obesity. The framework outlines how stigma operates at multiple socio-ecological levels (individual to policy) and identifies its drivers, facilitators and manifestations (Stangl et al. 2019).

Building on these conceptual foundations, stigma-reduction theories emphasise the need to address root causes and promote inclusion, drawing from psychological, social and behavioural disciplines. For example, Positive Psychology and Hope Theory encourage resilience and self-advocacy (Ysasi, Chen & Jones 2018), while Social-Psychological Theories explore the origins of stigma and social identity to inform interventions (Scior 2016). Relational Frame Theory applies behavioural techniques to enhance psychological flexibility and reduce harmful associations (Catrone & Koch 2021). Contact Theory highlights the importance of structured, meaningful intergroup interactions in reducing prejudice, cautioning that casual contact may reinforce stereotypes (Barney 2011).

Several systematic reviews have assessed stigma-reduction interventions, but most focus on health conditions or populations. For instance, Smythe, Adelson and Polack (2020) examined stigma against children with disabilities in LMICs (Smythe et al. 2020), while Jackson-Best and Edwards (2018) reviewed stigma related to HIV or AIDS, mental health and physical disability (Jackson-Best & Edwards 2018).

### Regional focus and purpose of the review

In Africa, stigma remains a major barrier to implementing the CRPD (Mostert 2016). It contributes to exclusion, low self-esteem and vulnerability to violence and abuse (Department for International Development 2018). For example, in Tanzania, stigma against people with albinism has led to harmful practices, social isolation and denial of basic rights. Addressing stigma is essential to improve quality of life of people with disabilities and ensure their safety and inclusion in society (Mostert 2016).

To promote inclusion, (non)government organisations and other institutions must understand the nature of stigma in their contexts and identify effective interventions tailored to different groups.

This review synthesises evidence on the effectiveness of interventions aimed at reducing stigma relating to any disability. The review centred on the evidence from sub-Saharan Africa and the following research questions:

- *Which interventions have been tested to reduce disability-related stigma in the studied settings?*
- *What was the reported effect of the evaluated interventions?*
- *What was the risk of bias of the studies reporting this evidence?*

## Research methods and design

The findings of this systematic review are presented adhering to the preferred reporting items for systematic review and meta-analysis (PRISMA) guidelines (S1 Table). The study protocol was not registered; however, it is available on request.

### Search strategy

Peer-reviewed literature was searched across multiple databases, including EBSCO, CINAHL, EMBASE, PsycINFO, ProQuest, PubMed, IDEA and ELDIS, up to 27 March 2025. Additionally, we searched for grey literature on relevant websites, screened references and consulted experts. Searches were limited to studies from 2006 onwards, in English, Portuguese and French, with no restrictions on publication status. Search terms included concepts related to disability, sub-Saharan and stigma (S2 Appendix 1).

### Inclusion criteria for the review

To be included in the review, articles were required to present primary data relevant to the research questions, regardless of the study design. The intervention had to measure changes in stigma as either a primary or secondary outcome. The target population was people with disabilities, as defined by the UNCRPD, including people with HIV or AIDS. The studies had to be conducted in sub-Saharan Africa and published after 2006. Additionally, the articles needed to be available in English, French or Portuguese.

### Screening

Two reviewers (Bhavisha Virendrakumar and Cathy Stephen or Vladimir Pente) independently screened abstracts, titles and full-text articles against the inclusion and exclusion criteria. Disagreements were resolved through discussion with a third reviewer (Emma Jolley). Studies were included if they met all the criteria specified in the section above.

### Data extraction and quality assessment

Two authors (Bhavisha Virendrakumar and Cathy Stephen or Vladimir Pente) independently extracted data and assessed the risk of bias of included studies. They piloted the data extraction form and used the Critical Appraisal Skills Programme (CASP) checklists for eight study designs. Because CASP checklists lack a uniform approach for comparing risk of bias across different designs, an aggregated risk of bias metric was conducted (methodology available upon request). Assessment criteria included methodology, findings and their validity and contextualisation. The appraisal tools were piloted with three studies to ensure validity and reliability. Risk of bias was categorised as follows:

- Low risk of bias – scoring profile of eight plus ‘yes’ answers.
- Medium risk of bias – scoring profile between six and seven ‘yes’ answers.
- High risk of bias – scoring profile of five or less ‘yes’ answers.

As per Smythe et al. (2020), the effectiveness of interventions was classified as positive (all stigma-related outcomes improved significantly), negative (all outcomes worsened significantly), ‘null’ (no significant change), or mixed (a mix of ‘positive’, ‘negative’ and ‘null’ changes across outcomes).

### Data synthesis

Findings are presented narratively, focusing on studies with medium and low risk of bias, to ensure greater confidence in the results. Understanding the design and implementation of interventions requires a coherent characterisation and underlying logic (Michie, Van Stralen & West 2011). Therefore, the review authors categorised interventions from the primary studies using the Behaviour Change Wheel framework (Michie et al. 2011), which comprises nine intervention functions and seven policy categories for classifying stigma-reducing interventions. Intervention functions aim to change behaviour, while policies represent actions by authorities that enable or support these interventions (Michie et al. 2011). Interventions include ‘education’, which aims to enhance familiarity; ‘persuasion’, which uses communication to evoke emotional responses or prompt action; ‘incentivisation’, which creates compensation expectations; and ‘coercion’, which introduces the anticipation of punishment or costs. ‘Training’ refers to developing skills, while ‘restriction’ involves applying rules to limit opportunities for the target behaviour or to encourage it by reducing opportunities for competing behaviours. ‘Environmental restructuring’ entails altering ‘physical or social settings’; ‘modelling’ involves setting examples for others to follow; and ‘enablement’ refers to increasing resources or lowering barriers to improve ability or prospect.

Policy actions include: ‘communication’ through media; ‘guidelines’ that recommend or require practices; ‘fiscal’ measures using taxes; ‘regulations’ that set behavioural rules; ‘legislation’ referring to the creation or amendment of laws; ‘environmental or social planning’ to shape surroundings; and ‘service provision’ referring to the delivery of services (Michie et al. 2011).

### Ethical considerations

This article followed all ethical standards for research without direct contact with human or animal subjects.

## Results

### Search results

**FIGURE 1:**
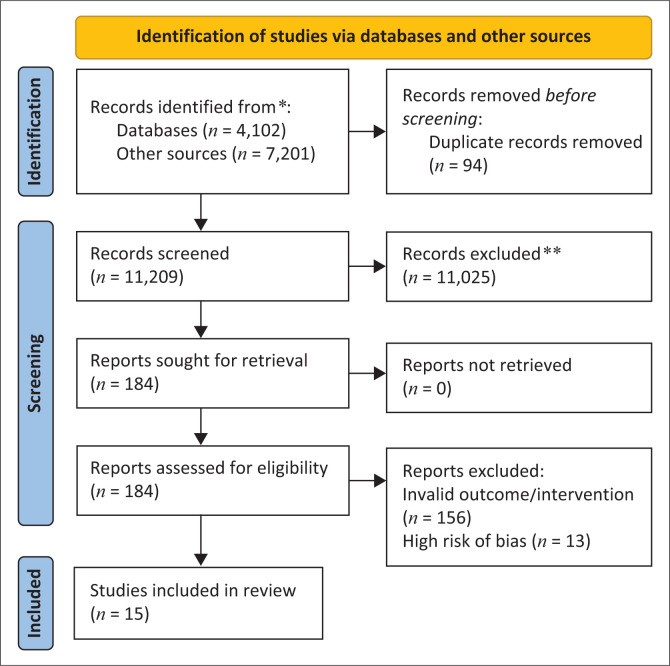
Search flow chart.

The search process identified 11 209 unique records. Following screening, 11 194 records were excluded because of the following reasons: they did not test a specific intervention, their outcomes did not measure changes in disability-related stigma, were published before 2006, or were conducted outside sub-Saharan Africa. Ultimately, 28 studies remained, all of which were in English. Of these, 13 studies were excluded because they were rated as ‘high risk of bias’ based on critical assessment, meaning their results and conclusions could not be deemed reliable. Consequently, 15 studies were included in the final review (Figure 1).

### Description of included studies

#### Geographic and impairment focus

Of the 15 included studies, four were conducted in Ethiopia (Asher et al. 2018; Hanlon et al. 2019; Tilahun et al. 2019; Tora et al. 2022), two each in Kenya (Mutiso et al. 2019; Odeny et al. 2013), Nigeria (Eze et al. 2015; Odukoya 2017), and Zambia (Hepperlen et al. 2021; Jurgensen et al. 2013), and one study each in Malawi (Creel et al. 2011), Rwanda (Creel et al. 2011), Uganda (Nabunya et al. 2024) and Ghana (Ben-Zeev et al. 2024). One study was a multi-country study conducted in Lesotho, Malawi, South Africa and Tanzania (Uys et al. 2009).

The studies examined stigma associated with various health conditions and impairments, including six studies on HIV or AIDS (Creel et al. 2011; Jurgensen et al. 2013; Michielsen et al. 2012; Nabunya et al. 2024; Odeny et al. 2013; Uys et al. 2009), four on mental health (Asher et al. 2018; Ben-Zeev et al. 2024; Hanlon et al. 2019; Mutiso et al. 2019) (schizophrenia, schizophrenia and bipolar disorder, various conditions and conditions unspecified), and one study each on epilepsy (Eze et al. 2015), autism (Tilahun et al. 2019), lower limb lymphoedema (Tora et al. 2022), and intellectual impairments (Odukoya 2017). In one study, the type of disability was not specified (Hepperlen et al. 2021).

#### Type of stigma

Two-thirds of the studies (10/15) failed to report the type of stigma addressed. The remaining five used terms such as individual (Jurgensen et al. 2013), enacted (Michielsen et al. 2012), perceived (Odeny et al. 2013), or internalised (Ben-Zeev et al. 2024), with one study addressing both internalised and anticipated stigma (Nabunya et al. 2024).

#### Study populations

The studies examined interventions targeting various population groups, including individuals with and without disabilities. Participants without disabilities included the general population (Asher et al. 2018; Creel et al. 2011; Hepperlen et al. 2021; Odukoya 2017; Tora et al. 2022), students (Michielsen et al. 2012) and professional groups such as nurses (Uys et al. 2009), community health workers (Mutiso et al. 2019), extension health workers (Tilahun et al. 2019), community-based rehabilitation workers (Asher et al. 2018) and teacher trainees (Eze et al. 2015). Studies involving participants with disabilities focused on individuals living with HIV or AIDS (Creel et al. 2011; Jurgensen et al. 2013; Michielsen et al. 2012; Nabunya et al. 2024; Odeny et al. 2013; Uys et al. 2009) and patients with schizophrenia or bipolar disorder, including one study addressing children with disabilities without further details (Asher et al. 2018; Ben-Zeev et al. 2024; Hanlon et al. 2019; Mutiso et al. 2019).

#### Study design

Studies used different designs, including five randomised controlled trials (RCTs) (Creel et al. 2011; Jurgensen et al. 2013; Michielsen et al. 2012; Nabunya et al. 2024; Odukoya 2017), one single-arm trial (Ben-Zeev et al. 2024), three before-and-after surveys (Eze et al. 2015; Hanlon et al. 2019; Hepperlen et al. 2021; Mutiso et al. 2019; Odeny et al. 2013), two post-intervention surveys (Tilahun et al. 2019; Uys et al. 2009), and one study each used a qualitative (Tora et al. 2022) and a mixed methods approach (Asher et al. 2018).

#### Stigma tools

Tools for measuring stigma varied and included Discrimination and Stigma Scale-12 (DISC-12) (Asher et al. 2018; Hanlon et al. 2019; Mutiso et al. 2019), HIV or AIDS self-administered Stigma instrument (Uys et al. 2009), Nyblade and MacQuarrie tool (Creel et al. 2011), Internalised Stigma of Mental Illness tool (ISMI-10) in combination with the Other as Shamer Scale (OAS) tool (Ben-Zeev et al. 2024) (*n* = 1), Intellectual Disabilities Literacy scale (Odukoya 2017) and Berger Stigma Scale (Nabunya et al. 2024). The remaining seven studies employed questionnaires, but failed to name the scale used (Eze et al. 2015; Hepperlen et al. 2021; Jurgensen et al. 2013; Michielsen et al. 2012; Odeny et al. 2013; Tilahun et al. 2019; Tora et al. 2022) (S3 Table).

### Types of interventions and policy action functions

**FIGURE 2:**
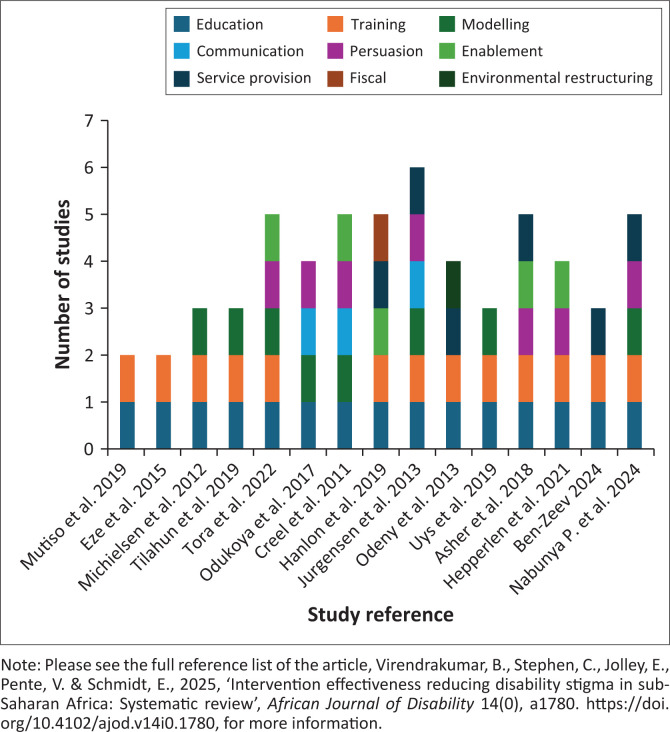
Intervention categories tested by included studies.

Studies employed two (*n* = 2), three (*n* = 4), four (*n* = 3), five (*n* = 5) or six (*n* = 1) intervention categories and/or policy actions from the Behaviour Change Wheel framework. Education was the most common intervention category (*n* = 15/15), followed by training (*n* = 13/15) and modelling (*n* = 8/15), often combined with education and training (*n* = 6/15). Communication was the most frequently employed function (*n* = 3/15) to support intervention delivery, followed by fiscal and environmental restructuring employed by one study each (Figure 2 and S4 Table).

#### Education

Education interventions, employed by all 15 studies, primarily involved group sessions with health professionals (Hanlon et al. 2019), patients (Odeny et al. 2013), parent peers (Nabunya et al. 2024), communities (Mutiso et al. 2019) and traditional healers, as well as a lecture (Eze et al. 2015); a film (Odukoya 2017), a radio programme (Creel et al. 2011), printed materials (Hepperlen et al. 2021; Tilahun et al. 2019; Tora et al. 2022); smartphone-delivered education content (Ben-Zeev et al. 2024); home-based discussions (Asher et al. 2018); counselling; and performing art sessions (e.g. drama, songs) (Michielsen et al. 2012).

#### Training

Skill development training sessions (10/15 studies) focused on health workers (Hanlon et al. 2019; Mutiso et al. 2019; Nabunya et al. 2024; Tora et al. 2022), student peer educators (Michielsen et al. 2012) and lay workers (Asher et al. 2018). Strategies included digital communication (Ben-Zeev et al. 2024); video-based training (Tilahun et al. 2019); practical clinical training (Hanlon et al. 2019; Mutiso et al. 2019); community sessions (Hepperlen et al. 2021); and lectures (Eze et al. 2015). Examples of targeted skills included integrated mental healthcare competencies, including diagnosis and treatment (Hanlon et al. 2019; Mutiso et al. 2019), peer-led health education competencies, such as communication and leadership skills (Michielsen et al. 2012), digital-supported collaborative mental healthcare competencies, such as digital literacy (Ben-Zeev et al. 2024), community-based rehabilitation delivery skills including counselling and communication (Asher et al. 2018), and community-based facilitation and health education skills, including community engagement and delivery of care (Tora et al. 2022).

#### Modelling

Modelling strategies (8/15 studies) involved healthcare professionals (Jurgensen et al. 2013; Nabunya et al. 2024; Tilahun et al. 2019; Uys et al. 2009), people with disabilities (Creel et al. 2011; Odukoya 2017; Tora et al. 2022), peer educators (Michielsen et al. 2012; Nabunya et al. 2024), and community members (Tora et al. 2022) as intervention facilitators to inspire others, encourage empathy and demonstrate positive behaviours for others to imitate.

#### Persuasion

Persuasion techniques (7/15 studies) were used to encourage treatment adherence (Nabunya et al. 2024), seek care and support (Asher et al. 2018; Hepperlen et al. 2021; Jurgensen et al. 2013; Tora et al. 2022) and address misconceptions to induce positive feelings about disability (Creel et al. 2011; Odukoya 2017).

#### Service provision

Different models of service delivery (6/15 studies) included Group Cognitive Behavioural Therapy (G-CBT) by trained parent peers (Nabunya et al. 2024), home-based counselling and testing services (Jurgensen et al. 2013), screening, outreach and social support from health extension workers (Hanlon et al. 2019), tailored services through a mobile application, nurse support (Ben-Zeev et al. 2024), provision of integrated health services (Odeny et al. 2013), home visits and community support groups (Asher et al. 2018).

#### Enablement

Enablement (5/15 studies), involved facilitating access to healthcare services through referral to health services (Asher et al. 2018), increasing awareness about the availability of treatment (Tora et al. 2022), enabling primary healthcare workers to provide first-line of treatment (Hanlon et al. 2019), creating opportunities for peer learning (Tora et al. 2022), developing basic skills and problem-solving techniques (Asher et al. 2018), facilitating access to information (Creel et al. 2011; Hepperlen et al. 2021), advocating for better community support (Hepperlen et al. 2021) and accessing support groups (Creel et al. 2011).

#### Communication

Three studies incorporated a communication function to support intervention delivery. Two studies utilised mass media, including radio, as part of the stigma-reduction intervention (Creel et al. 2011) and social media to recruit participants, reach a wide audience and facilitate information dissemination (Odukoya 2017). Another study combined print materials, electronic messages and radio broadcasts to promote the availability of healthcare services (Jurgensen et al. 2013).

#### Environmental restructuring

One study restructured the healthcare environment by integrating clinical spaces (pharmacy, health education, laboratory services) for people with HIV or AIDS (Odeny et al. 2013).

#### Fiscal

One study addressed financial barriers by providing medical support for medication supply management, including the establishment of a drug fund (Hanlon et al. 2019).

### Intervention combinations and their effectiveness

Out of 15 studies, eight reported a positive effect of interventions on reducing disability-related stigma (Ben-Zeev et al. 2024; Eze et al. 2015; Michielsen et al. 2012; Mutiso et al. 2019; Nabunya et al. 2024; Odukoya 2017; Tilahun et al. 2019; Tora et al. 2022). Five studies showed mixed effects (Asher et al. 2018; Creel et al. 2011; Hepperlen et al. 2021; Odeny et al. 2013; Uys et al. 2009), and two found no changes in disability-related stigma post-intervention (Hanlon et al. 2019; Jurgensen et al. 2013).

#### Studies that showed positive effects on stigma-related outcomes

**FIGURE 3:**
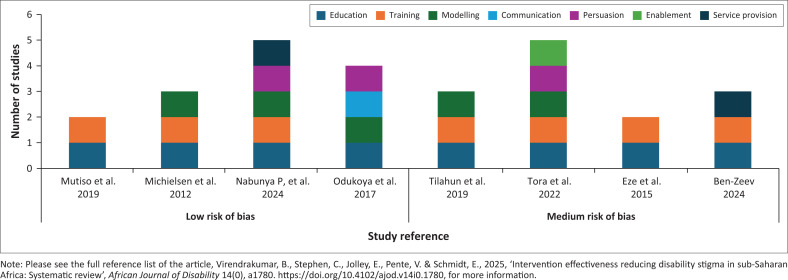
Studies that showed positive effects of intervention on stigma-related outcomes.

Out of eight studies that showed a positive effect, four were of low risk of bias and four were of medium risk of bias (Figure 3). Three studies that specified the type of stigma addressed, one focused on enacted stigma (Michielsen et al. 2012), one on internalised stigma (Ben-Zeev et al. 2024), and one on internalised and anticipated stigma (Nabunya et al. 2024). The remaining five studies did not report the type of stigma targeted but aimed to change attitudes, knowledge, beliefs, expectations, social distancing, unfair treatment and discrimination towards people with disabilities, the general population, teachers and health workers (Eze et al. 2015; Mutiso et al. 2019; Odukoya 2017; Tilahun et al. 2019; Tora et al. 2022). These studies implemented interventions comprising two to five components, each classified according to the Behaviour Change Wheel framework.

**Education and training:** Two studies evaluated the effects of education and training. A study from Nigeria evaluated the effect of a 1.5-h lecture on epilepsy with teacher trainees and found improved knowledge of the condition 12 weeks post-intervention (Eze et al. 2015). A study from Kenya assessed the effect of a 1-h community education session combined with a 5-day face-to-face training for faith and traditional healers, community health workers, and clinicians using the WHO Mental Health Treatment Gap Intervention Guidelines (mhGAP-IG) (Mutiso et al. 2019). At 6-month follow-up, there was a significant reduction in unfair treatment scores, particularly from sources closest to participants (e.g. neighbours, family) and a significant increase in overcoming stigma scores measured by the DISC-12 stigma tool (Mutiso et al. 2019).

**Education, training and modelling:** Two studies, one of low and one of medium risk of bias, showed a positive effect of interventions that combined education, training and modelling components (Michielsen et al. 2012; Tilahun et al. 2019). A study in Rwanda targeted enacted stigma among secondary school students, where peer educators conducted group and individual counselling sessions, organised drama performances and interactive activities to educate their fellow students about HIV or AIDS and model positive, responsible behaviours. Enacted stigma was measured using a self-administered questionnaire on two attitudes: refusal to be taught by an HIV-positive teacher and whether they thought students with HIV should be expelled from school. At 6-month follow-up, enacted stigma score reduced from 0.35 to 0.19 and was 0.24 at 12-month follow-up (Michielsen et al. 2012).

In Ethiopia, two interventions (a basic mental health training module and a training supplemented by a video and educational resources on early detection and counselling) were evaluated to assess their impact on autism-related stigma among rural health workers. Both intervention groups reported fewer negative beliefs and lower social distancing compared to the control group, with the enhanced intervention group showing a greater effect. However, the enhanced group exhibited lower positive expectations of children with autism compared to the control group (Tilahun et al. 2019).

**Education, training and service provision:** A study from Ghana (medium risk of bias) evaluated the effectiveness of an intervention designed for healers supporting individuals with mental illnesses. The intervention included the M-Healer toolkit, an Android-based application offering psycho-education, patient tracking, notifications and monitoring. Healers were trained to use the application through interactive sessions (e.g. demonstrations and role-plays) and received continued support from a nurse to ensure effective implementation (Ben-Zeev et al. 2024). Additionally, a registered mental health nurse, trained in the WHO’s mhGAP-IG package, administered pharmacotherapy, monitored health metrics and conducted weekly assessments, with support from study psychiatrists through telecommunication and weekly meetings. The findings indicated a reduction in feelings of shame, with the OAS scores decreasing from 41.9 at baseline to 28.5 at endline, and a decrease in internalised stigma, with ISMI-10 scores dropping from 30.4 to 24.9 over the same period (Ben-Zeev et al. 2024).

**Education, modelling, persuasion and communication:** A low risk of bias study from Nigeria evaluated an intervention targeting the general population aged 18 and above, recruited through social media, which combined education, communication, modelling and persuasion strategies (Odukoya 2017). The intervention featured a 6-min film portraying individuals with intellectual disabilities, using emotional narratives to highlight their capabilities. Data were collected at three time points, and stigma was assessed across five attitudinal domains spanning affect (e.g. discomfort, sensitivity), cognition (e.g. understanding causes, abilities, and rights) and behaviour (e.g. interacting with people with intellectual impairments). The Intellectual Disabilities Literacy scale was also used to assess causal beliefs. The intervention group demonstrated significant immediate improvements in discomfort, sensitivity and behavioural attitudes, although these effects diminished after 1 month. Knowledge of rights improved initially but showed no sustained change, while beliefs about capacities improved and remained positive. Superstitious beliefs significantly declined, but only at the 1-month follow-up.

**Education, training, modelling, enablement and persuasion:** A qualitative study from Ethiopia explored the impact of an intervention that combined education, training, modelling, enablement and persuasion through community conversations to enhance health literacy about lower limb lymphoedema. The intervention engaged 400 participants and facilitators, including religious leaders and patients serving as role models, who were trained using a facilitation guide outlining principles and methods for effective health communication. Sessions with patients incorporated interactive techniques such as role play and storytelling, functioning as self-help groups, and were complemented by broader community health information. Focus group discussions and interviews revealed self-reported improvements in health literacy, reductions in misconceptions, increased adherence to clinical self-care and decreased self-stigma (Tora et al. 2022).

**Education, training, modelling, persuasion and service provision:** A study from Uganda (low risk of bias), addressing anticipated and internalised stigma, tested two different interventions to support adolescents with HIV: Cognitive Behavioural Therapy (G-CBT) and Multiple Family Group-Based Family Strengthening (MFG-FS). The G-CBT consisted of 10 biweekly 1-h sessions delivered by two trained health counsellors for adolescents with HIV, focusing on psycho-education, cognitive restructuring and skill-building. The MFG-FS intervention consisted of 10 biweekly sessions delivered by two trained parent peers for families (adolescents with HIV and their caregivers), focusing on communication, emotional support and problem-solving. Based on the Berger stigma scale, the authors found significant effects on both internalised and anticipated stigma when compared to usual care (Nabunya et al. 2024).

#### Studies that showed mixed effect on stigma-related outcomes

**FIGURE 4:**
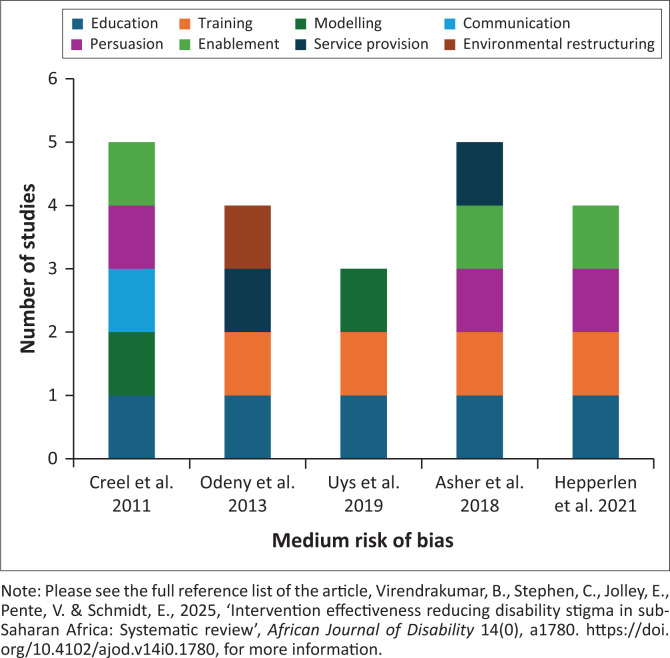
Studies that showed mixed effect of interventions on stigma-related outcomes.

Five studies, all with medium risk of bias, showed mixed effect of interventions, indicating that some interventions may work while others may not change or change outcomes for the worse (Figure 4). One study explicitly reported addressing perceived stigma (Odeny et al. 2013), while the remaining four studies failed to report the type of stigma addressed (Asher et al. 2018; Creel et al. 2011; Hepperlen et al. 2021; Uys et al. 2009). Two of these four studies aimed at reducing stigma among people with and without disabilities. The studies included between three and five behaviour change components based on the Behaviour Change Wheel categorisation.

**Education, training and modelling:** A study addressing HIV or AIDS-related stigma covered multiple countries, including Lesotho, Malawi, South Africa, Swaziland and Tanzania (Uys et al. 2009). The intervention paired a team of nurses and people with HIV or AIDS to share information on the impact of stigma, plan and implement stigma-reducing activities, and identify positive role models. Among people with HIV or AIDS, within a month after the intervention, cumulative results for all countries showed a significant reduction in the overall perceived, workplace, and self-stigma. No changes were noted among nurses in stigma or psycho-social measures, though HIV testing uptake among nurses increased significantly by project completion.

**Education, training, persuasion and enablement:** A medium risk of bias study in Zambia addressed community stigma towards children with disabilities. The programme employed drama, singing, dancing, photography exhibitions, playgroup parents’ interactions and church presentations to educate and dispel negative beliefs about children with disabilities. Community health workers, church leaders, parents and playgroup facilitators were trained to deliver interventions. Changes in stigma were assessed at 18-month post-intervention, with lower levels reported by families of children with disabilities, but no changes in community members’ attitudes (Hepperlen et al. 2021).

**Education, training, persuasion, enablement and service provision:** A mixed methods study from Ethiopia integrated a stigma-reduction intervention into a community-based rehabilitation (CBR) programme. The intervention involved continued support from CBR workers (CBRWs) for 10 people with schizophrenia and their families or carers, aiming to address stigma within the context of community care and support.

Community-based rehabilitation workers received a 5-week training and provided support over 12 months through home visits, psycho-education and counselling on returning to work, with an average of 21 sessions. Additionally, six community members were mobilised to offer financial and practical support to the same families. Stigma was assessed using the DISC-12 tool at baseline, 6 months and 12 months, while qualitative data were gathered through interviews and focus group discussions at 2 months and 12 months. The intervention was generally well-received, fostering trust among participants, carers and CBRWs. However, some participants perceived home visits as stigmatising, and there were concerns regarding treatment recommendations. Notably, CBRWs’ attitudes towards people with schizophrenia improved, community support was positively received, and the number of participants reporting stigma declined from seven to two (Asher et al. 2018).

**Education, training, service provision and environmental restructuring:** A study in Kenya integrated HIV or AIDS care into primary health services to reduce perceived stigma faced by people with HIV or AIDS and improve patient satisfaction. The integrated model allowed for any-day health care visits instead of specific days separate from other patients. Regardless of HIV status, all patients received health education together in a shared waiting area; medications were dispensed from the same pharmacy; HIV-related and other medical tests were conducted in the same laboratory; and clinical staff were trained on HIV and non-HIV topics at the same time. At 3-month and 12-month post-intervention, findings showed high service satisfaction throughout. Views on equitable treatment improved over time for men but not women, and discomfort levels varied: men’s discomfort decreased temporarily, while women’s discomfort increased consistently (Odeny et al. 2013).

**Education, modelling, persuasion, enablement and communication:** A study from Malawi used social marketing interventions to address HIV or AIDS-related stigma through radio programmes. Participants were divided into three groups: one group listened to ‘Radio Diaries’ (RD), where an HIV-positive individual shared 20-min diary entries about their experiences with health services and coping with the condition. The second group (RD + D) also listened to these diary entries, as well as call-in shows and expert panel discussions. The control group listened to a radio programme of similar length on child labour.

These episodes were broadcast on six local radio stations, and the villages involved in the study were randomly assigned to one of the groups. Post-intervention, results showed the RD group had lower fear of contact and shame compared to the control group, while the RD + D group showed mixed results. There were no differences in willingness to disclose HIV status among the groups (Creel et al. 2011).

#### Studies that showed null effect of stigma-related outcomes

Two low risk of bias studies, addressing self-stigma among people with HIV or AIDS and experienced and perceived discrimination among patients with psychiatric disorders, showed null effects of interventions on stigma-related outcomes.

**Education, training, enablement, fiscal and service provision:** A study conducted in rural Ethiopia evaluated the impact of a district-level plan for task-shared mental healthcare, which included training and supervision of primary healthcare (PHC) workers alongside community-level support through awareness-raising, community mobilisation and CBR. Using the DISC-12 tool, no significant changes in experienced stigma were observed between baseline and 6 months. However, a modest reduction in perceived discrimination was noted between the 6- and 12-month follow-ups (Hanlon et al. 2019).

**Education, training, modelling, persuasion, service provision and communication:** An RCT in rural Zambia addressed stigma among people with HIV or AIDS in 18 villages through home-based voluntary counselling and testing (VCT) provided by trained lay counsellors, supplemented by community mobilisation. Another 18 villages had standard facility-based VCT. Stigma was measured using questionnaires 18 months post-intervention. The findings showed reductions in stigma related to quality of care and verbal abuse in both groups, with no significant differences between them (Jurgensen et al. 2013).

## Discussion

To the best of our knowledge, this is the first systematic review of stigma-reducing interventions targeting different population groups with diverse disabilities in sub-Saharan Africa. The findings provide interesting insights for future disability focused programmes and stigma research.

From a Critical Disability Theory (CDT) perspective, the limited number of interventions and evaluations identified in this review reflects systemic marginalisation of disability within global health and development agendas, and the dominance of medical or charitable models over rights-based approaches (Gillies 2014). Most studies in this review focused on HIV or AIDS, likely because of sustained international development funding over the past few decades. However, this focus may inadvertently reinforce hierarchies of disability and overlook the varied experiences of people with other impairments (Gillies 2014). Critical Disability Theory urges scrutiny of such funding patterns and calls for inclusive research that centres the voices of people with disabilities (Gillies 2014).

Overall, the findings of this review imply that while multicomponent interventions are common, their effectiveness for reducing stigma is variable and context-dependent.

Many interventions included education and training components, but their small scale and short duration raise concerns about sustainability and structural impact. Additionally, educational and training initiatives alone may not adequately address the social drivers of stigma, such as power imbalances and exclusionary practices (Gillies 2014). Interventions that also include components like modelling (role models), persuasion (emotional engagement) and enablement (reducing barriers) may be more impactful, but only if tailored to local needs.

Furthermore, the lack of clear theories of change and rationale for targeting specific groups in most studies limits the ability to identify what works, for whom, and why. The inconsistent use of stigma measurement tools and poor reporting of intervention logic make it difficult to compare studies or draw firm conclusions about effectiveness. The lack of clear theories of change and inconsistent stigma measurement tools among the included studies may reflect the limited involvement of people with disabilities in intervention design and evaluation. CDT emphasises the need to recognise their expertise and ensure their meaningful participation in shaping research agendas (Gillies 2014).

Finally, the methodological quality of included studies was generally low. Poor reporting and design limitations were common, echoing findings from other reviews (e.g. Smythe et al. 2020). Strengthening methodological rigour and transparency is urgently needed.

Based on the findings of our systematic review and supported by the wider literature, future research should clearly define stigma and reflect on whose perspectives shape the research agenda (Corcuff et al. 2025). People with disabilities should be engaged as co-researchers, with attention to intersectionality (Wickenden 2023). Measurement tools must be culturally adapted and validated, with transparent reporting of development processes (Werner 2016). Mixed methods and longitudinal designs can better capture the complexity and sustainability of stigma reduction (Plano Clark et al. 2015). Frameworks like Stangl et al. (2019) can support consistent conceptualisation and design, but CDT cautions against approaches that ignore social and political contexts (Gillies 2014). A combination of stigma-related frameworks and stigma-reduction theories may offer a rich foundation for designing stigma-reduction strategies that are both conceptually sound and practically effective.

It is also important to note that publication bias may have affected the number of studies available for our review, which further underscores the need to encourage programme implementers to publish their stigma evaluations irrespective of study findings. This more transparent approach can help governments and donors save their limited resources and prioritise interventions that work. Further high-quality research is urgently needed to understand how different interventions work independently or in combination.

### Limitations of the review

Limitations should be noted when considering the findings of this review. Most interventions focused on stigma related to HIV or AIDS or mental health, likely because of funding availability. This review, while thorough, may be prone to publication bias because of language restrictions and the tendency to publish positive results.

## Conclusion

This systematic review has demonstrated the limited availability of high-quality research testing the impact of interventions to reduce disability stigma in sub-Saharan Africa. Challenges exist in both the articulation of stigma concepts and the proven effectiveness of different combinations of interventions. This is further complicated by the wide range of target groups, settings and types of interventions used to reduce stigma, and the diverse metrics used to measure and demonstrate changes in stigma.

Effective strategies are under-researched despite stigma’s global impact. Stakeholders should: (1) invest in comprehensive evaluations with a clear framework and consistent metrics, (2) be transparent about intervention choices and target groups, and (3) publish all results, whether positive or negative.
